# Age Encoded Adversarial Learning for Pediatric CT Segmentation

**DOI:** 10.3390/bioengineering11040319

**Published:** 2024-03-27

**Authors:** Saba Heidari Gheshlaghi, Chi Nok Enoch Kan, Taly Gilat Schmidt, Dong Hye Ye

**Affiliations:** 1 Department of Computer Science, Marquette University, Milwaukee, WI 53233, USA; 2 Department of Electrical and Computer Engineering, Marquette University, Milwaukee, WI 53233, USA; kanxx030@gmail.com; 3 Department of Biomedical Engineering, Marquette University and Medical College of Wisconsin, Milwaukee, WI 53233, USA; tal.gilat-schmidt@marquette.edu; 4 Department of Computer Science, Georgia State University, Atlanta, GA 30303, USA

**Keywords:** generative adversarial networks, medical image segmentation, organ segmentation

## Abstract

Organ segmentation from CT images is critical in the early diagnosis of diseases, progress monitoring, pre-operative planning, radiation therapy planning, and CT dose estimation. However, data limitation remains one of the main challenges in medical image segmentation tasks. This challenge is particularly huge in pediatric CT segmentation due to children’s heightened sensitivity to radiation. In order to address this issue, we propose a novel segmentation framework with a built-in auxiliary classifier generative adversarial network (ACGAN) that conditions age, simultaneously generating additional features during training. The proposed conditional feature generation segmentation network (CFG-SegNet) was trained on a single loss function and used 2.5D segmentation batches. Our experiment was performed on a dataset with 359 subjects (180 male and 179 female) aged from 5 days to 16 years and a mean age of 7 years. CFG-SegNet achieved an average segmentation accuracy of 0.681 dice similarity coefficient (DSC) on the prostate, 0.619 DSC on the uterus, 0.912 DSC on the liver, and 0.832 DSC on the heart with four-fold cross-validation. We compared the segmentation accuracy of our proposed method with previously published U-Net results, and our network improved the segmentation accuracy by 2.7%, 2.6%, 2.8%, and 3.4% for the prostate, uterus, liver, and heart, respectively. The results indicate that our high-performing segmentation framework can more precisely segment organs when limited training images are available.

## 1. Introduction

Deep learning has played critical roles in various applications such as signal processing [1,2], image recognition [3,4], text classification [5], and image segmentation [6,7,8]. Medical imaging is one of the popular real-life applications of deep learning. Deep learning-based medical imaging techniques are proven to be more efficient than other approaches in clinical tasks [9,10,11]. One of the major applications of AI within the field of medical imaging is diagnostic radiology. Abdominal imaging is one of the essential sub-fields of diagnostic radiology. It is tied to crucial clinical applications such as computer-aided diagnosis, treatment planning, morphology, and organ-specific dose estimation. Abdominal multi-organ segmentation outlines essential organs, such as the heart, liver, bladder, prostate/uterus, and pancreas, by either computed tomography (CT) or magnetic resonance imaging (MRI). The precise annotation of organ boundaries is vital for patient safety and treatment. However, this process can be tedious when radiologists have to manually annotate each organ in patients [12].

Computed tomography (CT) was first invented in the early 1970s, and its clinical utilization grew rapidly in the following years [13,14]. CT imaging is a computerized tomographic version of X-ray imaging that has been widely used in diagnosing diseases and treatment planning, such as COVID-19 diagnosis [15], brain lesion detection [16], and organ-specific dose estimation. The CT imaging technique is a painless, fast, and non-invasive method that yields detailed images of various body organs for diagnostic purposes. CT images are widely used for radiation therapy and pre-operative planning, and accurate abdominal organ segmentation is essential in this area. However, the accuracy of abdominal organ segmentation remains challenging, especially in children, since children’s organs are hard to detect and are susceptible to ionizing radiation. The uterus and the prostate are some of the most radiosensitive abdominal organs. This is why CT is not a standard diagnostic imaging technique for reproductive organs in children. Hence, very few labeled datasets contain large amounts of pediatric reproductive organs. Therefore, the segmentation performances of state-of-the-art deep neural networks on these organs are often poor.

Manual segmentation is a labor-intensive and impractical task; as a result, different automated and semi-automated approaches for segmentation have been proposed for both pixelwise (2D) and volumetric (3D) segmentation. Deep learning methods such as U-Net [17], 3D U-Net [18], CE-Net [19], and Dense V-Net [20] are prevalent in medical image semantic segmentation. These networks have shown promising results in organ segmentation and are generally considered state-of-the-art. However, they all depend on large amounts of training data to achieve high segmentation accuracies.

Taly et al. [21] combined dose maps and organ segmentation masks to rapidly quantify CT doses. Their study extracts CT dose maps from Monte Carlo-based simulations, and a U-Net is used for organ segmentation. Jackson et al. [22] used a CNN with 3D convolutional layers to predict right and left kidney segmentation masks and coupled them with volumetric dose maps for organ dose estimation. Fang et al. [23] introduced a 2D-to-3D segmentation framework for CT organ segmentation. In this framework, the author increased the performance by jointly optimizing and transforming the 2D coarse result into 3D segmentation masks of coarse to fine. Okada et al. [24] used a statistical prediction-based atlas with modification on the distribution of CT values for each organ to segment upper abdominal organs. Their method was tested on eight abdominal organs, and the experimental results have shown the method’s ability to improve segmentation accuracy. Tong et al. [25] improved multi-organ segmentation performance by using a self-paced DenseNet. Their research combines learning-based attention mechanisms and dense blocks to improve the efficiency of the original DenseNet. Balagopal et al. [26] used a multi-channel 2D U-Net followed by a 3D U-Net to segment male pelvic CT images. They applied their 2D–3D hybrid network to a pelvic CT image dataset with 136 patients and reported the segmentation results on the test set. Zhou et al. [11] used a fully convolutional network (FCN) [27] and a V-Net to construct their segmentation network. The authors divided CT images into small patches and trained the two networks to segment 2D and 3D images, respectively. This research segmented 17 types of organs from a dataset with 240 CT scans. Gibson et al. [28] proposed a registration-free deep learning segmentation method and compared their results with a multi-atlas label fusion-based method to highlight their improvement in segmentation accuracy. They used dense V-Net/FCN networks to segment eight abdominal organs and validate the trained networks with a separate dataset with 90 patients. Alsamadony et al. [29] used a transfer learning approach to map low-resolution CT images to high-resolution CT images to reduce the patient’s exposure times. The authors used very deep super-resolution (VDSR) and U-Net to improve image quality. The authors compared the average peak signal-to-noise ratio (PSNR) values produced by both networks on a validation set with 400 images. The U-Net outperformed VDSR in their study, with improved image quality.

All the studies above cover adult organ segmentation, which is considered less challenging than segmenting pediatric organs. Moreover, the performance of the deep learning models highly depends on the size of the training dataset. Networks trained on small datasets are prone to overfitting and often generalize poorly in testing [30]. This paper proposes a method that generates new synthetic images using an age auxiliary classifier Pix2Pix (Age-ACP2P) while training a segmentation network. Our approach shows promising results in segmenting pediatric abdominal organs.

## 2. Methodology

Unet was first introduced by Ronneberger et al. [17] in 2015, and since then, it has been one of the most powerful networks in biomedical image segmentation. The Unet architecture is a symmetric U-shape that consists of two paths. The encoder path captures the context in the image, and the ecoder path transfers the latent features to the segmentation masks rather than the original image. Although Unet is widely used in medical image segmentation, it has limitations in extracting complex features or when there is a scarcity of annotated data for training. These limitations can hurt the segmentation accuracy [31]. Different techniques have been proposed in past years to tackle this issue, and adversarial learning has shown great potential.

**Figure 1:**
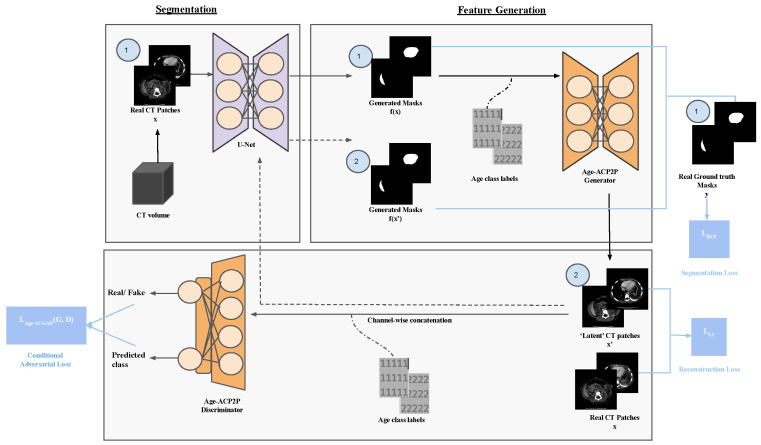
An overview of our proposed CFG-SegNet framework: First, we center-crop patches of abdominal CT images (denoted as 1) and run a forward pass through a U-Net to produce their corresponding segmentation masks. Age-ACP2P’s generator network subsequently uses these segmentation masks to reconstruct latent CT patches (denoted as 2). We then use the U-Net to segment these reconstructed patches to obtain a second set of segmentation masks. We expect the quality of the latent patches and segmentation masks will improve over time, given our novel loss function, which is a weighted sum of segmentation, reconstruction, and adversarial losses.

In this study, we proposed the CFG-SegNet that effectively segments CT images while generating new synthetic data during training. Figure 1 shows an overview of our proposed method. Our proposed framework consists of two netlists of the U-Net segmentation network and a feature-generating Age-ACP2P network. In a given training step, the U-Net generates a segmentation mask; the sector is translated into the latent feature by Age-ACP2P’s generator. The translated element is then used to retrain the U-Net, and this process continues until the loss converges. A novel loss function that combines segmentation and adversarial losses is used to jointly train a conditional GAN (cGAN) along with a segmentation network. We chose age auxiliary classifier Pix2Pix (Age-ACP2P) as our cGAN since previous work by Kan et al. [32] demonstrated its effectiveness in generating realistic age-conditioned CT images from their segmentation masks. As both networks are trained jointly, we expect the segmentation accuracy to improve over time. It is worth noting that Age-ACP2P was not used in testing, as we only evaluated the segmentation performance of the U-Net.

In the following sections, we will describe the generative adversarial networks, which represent the backbone of our proposed method, and give details of our proposed CFG-SegNet.

### 2.1. Generative Adversarial Networks (GANs)

Generative adversarial networks (GANs) were first introduced by Goodfellow et al. [33] in 2014, and GANs received lots of attention recently due to their ability to generate and synthesize realistic images from white noise vectors. GAN architecture consists of two competing networks: a generator network, *G*, and a discriminator network, *D* [34]. *G* takes a random noise vector, z, as an input and transforms it into an image, G(z). The discriminator, *D*, then attempts to maximize the log probability of assigning correct labels to both the real training images and synthetic images generated by *G*. This log probability can be expressed mathematically as

(1) log(D(z))+log(1−D(G(z))

On the other hand, *G* is trained to minimize the inverted log probability of *D*′s prediction of fake images log(1−D(G(z)). Since it is hard to minimize the inverted log probability log(1−D(G(z)) in practice, we seek to maximize D(G(z)) instead. Overall, the objective function of GAN can be formulated as a minimax loss:

(2) $$\arg\min_{G}\max_{D}L_{GAN}\left(G,D\right)=E_{x∼p_{data}\left(x\right)}+\left[logD\left(x\right)\right]+E_{z∼p_{z}\left(z\right)}\left[log\left(1-D\left(G\left(z\right)\right)\right)\right]$$

### 2.2. CFG-SegNet

Although the original GAN is capable of synthesizing realistic images, it can only synthesize the images in a random way and is often vulnerable to mode collapse. Mode collapse occurs when the generator chooses only to use the most accessible class to fool the discriminator. This behavior results in a lack of diversity in the synthesized images; hence, the network is more vulnerable to overfitting. In practice, mode collapse often happens due to class imbalance in training data.

One of the common ways to tackle the mode collapse issue is to incorporate side information and add conditions to a GAN’s generator. Conditional GAN (cGAN) [35] is a common type of GAN that uses a generator that conditionally generates images based on class labels. Adding conditions to the generator not only helps solve the mode collapse issue but also can improve training stability and generate images with better quality.

Our proposed CFG-SegNet uses a variant of GAN called the Pix2Pix, which is a type of conditional GAN designed for general image-to-image translation. Pix2Pix is built based on U-Net and uses adversarial learning to reach the modality transfer. In pix2pix, the generator is usually a U-Net, and the discriminator is a convolutional classifier. The loss function of Pix2Pix is an extension of conditional adversarial ($L_{cGAN}\left(G,D\right)$) and reconstruction ($L_{L1}$) losses:

(3) $$G^{*}=\arg\min_{G}\max_{D}L_{cGAN}\left(G,D\right)+\lambda L_{L1}$$

We can replace the first adversarial loss term, $L_{cGAN}\left(G,D\right)$, with the adversarial loss from auxiliary classifier GANs (ACGANs) to incorporate side information from image labels. The discriminator in ACGAN also produces a probability distribution, P(C|X)=D(X), over the class labels of the images, as well as producing a probability distribution, P(S|X)=D(X), over the image sources. Therefore, the objective function of ACGAN is defined as the log-likelihood of the correct source, $L_{S}$, and the log-likelihood of the correct class, $L_{C}$, where

(4) $$L_{S}=E\left[\log P(S=real|X_{real})\right]+E\left[\log P(S=fake|X_{fake})\right]$$

(5) $$L_{C}=E\left[\log P(C=c|X_{real})\right]+E\left[\log P(C=c|X_{fake})\right]$$

Since our study primarily focuses on the age of the patients, we employ a variant of ACGAN known as the Age-ACGAN to incorporate age information in CFG-SegNet. Age-ACGAN uses a slightly modified objective function of the ACGAN to compute the log-likelihoods of the correct image source ($L_{s}$) and the correct age class ($L_{a}$):

(6) $$L_{s}=E\left[\log P(S_{CT}=real|X_{real})\right]+E\left[\log P(S_{CT}=fake|X_{fake})\right]$$

(7) $$L_{a}=E\left[\log P(C_{age}=age|X_{real})\right]+E\left[\log P(C_{age}=age|X_{fake})\right]$$

Age-ACGAN’s discriminator attempts to maximize $L_{a}+L_{s}$, which means the log-likelihoods of assigning the correct source of a CT image, $CT_{source}$, and its respective age class label, $CT_{age}$, are always maximized. By denoting (4) and (5) as a single minimax loss term, $L_{age-ACGAN}$, and substituting it into (3), we obtain the objective function of Age-ACP2P:

(8) $$G^{*}=\arg\min_{G}\max_{D}L_{Age-ACGAN}\left(G,D\right)+\lambda_{L1}L_{L1}$$

Finally, we incorporate binary cross-entropy (BCE) loss into our combined loss function (5) to get the final objective function:

(9) $$G^{*}=\arg\min_{G}\max_{D}L_{age-ACGAN}\left(G,D\right)+\lambda_{L1}L_{L1}+\lambda_{BCE}L_{BCE}$$

Our final objective function has two tunable λ parameters, which control the weighting of the reconstruction and segmentation losses, respectively. If $\lambda_{BCE}$ is 0, we end up with Age-ACP2P’s objective function. The balance between the $L_{1}$ and BCE losses plays a critical role in the performance of CFG-SegNet. The $L_{1}$ loss ensures that the generated features maintain structural integrity and the details necessary for accurate segmentation. On the other hand, the BCE loss focuses on minimizing the difference between the predicted segmentation masks and the ground truth, ensuring high segmentation accuracy.

The co-dependent relationship between the segmentation network and the cGAN allows CFG-SegNet to effectively generate new data for training in each iteration of the training loop. At the beginning of training, segmentation masks are first generated from a forward pass through the segmentation network. These segmentation masks are subsequently translated back into the original image domain via an Age-ACP2P network. An Age-ACP2P network is a Pix2Pix combined with an Age-ACGAN (age auxiliary classifier GAN). Age-ACGAN was previously used to synthesize pediatric abdominal CTs conditionally, containing the pancreas. Similar to Age-ACGAN, age information is incorporated in Age-ACP2P by attaching an additional auxiliary classifier to its discriminator and by the channel-wise concatenation of age class labels to its inputs. We have enhanced the Unet training process by incorporating traditional data augmentation techniques, such as rotation and flip. Our proposed method offers a significant advantage over conventional augmentation methods. Unlike traditional techniques that simply apply the same image to the dataset, our approach generates new data and seamlessly integrates them. By doing so, we effectively reduce overfitting and prevent the repetition of identical data instances.

## 3. Dataset

**Figure 2:**
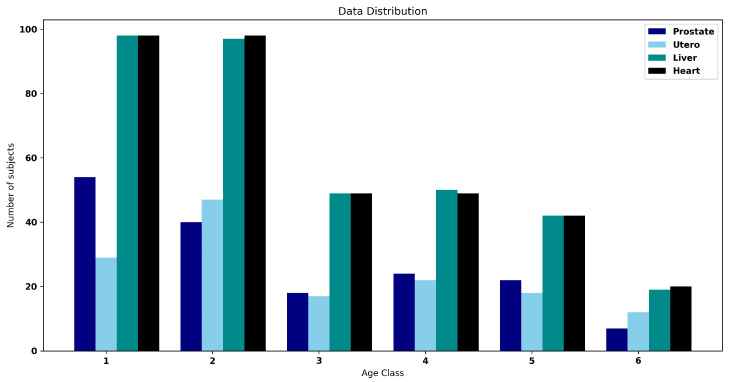
The number of subjects for each age class. Class 1 shows subjects aged 0 to 3 years; class 2 shows those between 4 to 6 years; class 3 shows those between 7 to 9 years; class 4 shows those between 10 to 12 years; class 5 shows those between 13 to 15 years; class 6 shows those with an age more than age 16 years.

This study uses the first version of pediatric chest/abdomen/pelvic CT exams with expert organ contours (Pediatric-CT-SEG) as our main dataset [36]. This dataset consists of 359 subjects (180 male and 179 female) aged from 5 days to 16 years and a mean age of 7 years. This dataset contains various chest/abdomen/pelvic CT scans, and in this research, we use CFG-SegNet to segment four organs (prostate, uterus, liver, and heart). It is worth mentioning that the availability of expert contours for these reproductive organs in this dataset is relatively lower than other organs because of the difficulty in visualizing these organs in pediatric CT images. Therefore, the segmentation of these organs is challenged by both the difficulty of organ localization and the reduced number of datasets. Our study includes the uterus and prostate, which are organs that are excluded from the V-net study due to these challenges [37]. In this dataset, there is a total of 165 subjects with prostate contours, 145 subjects with uterus contours, 355 subjects with liver contours, and 256 subjects with heart contours. In Figure 2, we show the data age distribution for each of the organs mentioned above.

The CT images in this dataset are stored in digital imaging and communications in medicine (DICOM) format, and the patients’ information is saved in DICOM headers. In order to pre-process the data, all our experiment images were center-cropped around the organ region, and we used slices with organ contour information. For the prostate and uterus, the final image size is 256×256, and for the liver and heart, the images are 512×512.

## 4. Experiment

This study conducted automated organ segmentation on CT images. Since data limitation is one of the significant difficulties in applying deep learning to medical images, we propose a novel segmentation framework with a built-in ACGAN that conditions age. Our proposed method simultaneously generates additional features during training to tackle the data limitation issue and help our network achieve higher segmentation accuracy. In order to test and validate our proposed network’s ability to conditionally generate CT images and the ability to improve organ segmentation, we compare the segmentation accuracy of our CFG-SegNet and one of the most common medical segmentation networks (U-Net network). In addition, we compare our method with the state-of-the-art GAN-based CutMix augmentation method, which cuts and pastes patches in training while labels are mixed proportionally [38]. CutMix blends the features and labels of different images and offers a unique approach to data augmentation, promoting the learning of robust and localizable features. Its applications are broad, ranging from general computer vision tasks to specialized domains, such as medical imaging, making it an essential tool for practitioners in the field of deep learning and artificial intelligence.

In this experiment, we used 70% of the data for training, 10% for validation, and 20% for testing. We only used image slices with a corresponding ground truth label, and there is no overlap between subjects in the training, testing, and validation sets. In this study, U-Net and CFG-SegNet were trained for 50 epochs using an Adam optimizer and an initial learning rate of 0.0002. In the training and testing phases, age class labels were concatenated to random Gaussian noise vectors, *z*, before Age-ACGAN’s generator and discriminator input. Cross-entropy was used in our implementation to calculate Age-ACGAN’s loss terms, $L_{s}$ and $L_{A}$, as described in Equations (6) and (7). The best validation weights were saved and used for evaluation on the test set. We validated our proposed method’s effectiveness by computing the dice similarity coefficient (DSC) between the segmentations to tackle the data limitation issue and help our network-fold cross-validation for the test set [39,40]. DSC is calculated using the following equation:

(10) $$DSC=\frac{2\times |X\cap Y|}{\left|X|+|Y\right|}$$

where *X* represents the set of pixels in the ground truth segmentation, and *Y* represents the set of pixels in the predicted segmentation. DSC is a measure of overlap between the two segmentation results, with a value ranging from 0 (no overlap) to 1 (perfect overlap).

### 4.1. Implementation Details

In this research, CFG-SegNet simultaneously generates novel training issues while learning the organ segmentation task over time. As a pre-processing step, the affine transformation was used to center the target organs. Affine transformations play an important role in medical image pre-processing, helping researchers analyze medical imaging data efficiently. Affine transformations are extensively utilized for registering images and aligning data into a common co-ordinate system, which gives the DL models better performance and increases the model’s reliability. Affine transformation integrates several transformations, including translation, rotation, scaling, and shearing, as noted in [41,42]. This combination of transformations allows for the imposition of geometric constraints. Consequently, it helps narrow down the search space and improve performance, providing a framework that is particularly beneficial for the deformable registration process. Given that the inputs in this case are not centered patches, applying affine transformations is essential for image alignment, resulting in inputs that are not only robust but also conducive to more accurate and reliable analyses. The main hurdle in the generation of synthetic images not only maintains a high degree of realism but also encapsulates the vast diversity found in various age groups, a critical factor for ensuring the applicability and accuracy of our model. Achieving a stable model that converges during the training process is another challenge that is necessary for the parameter tuning and training strategies to avoid overfitting. Furthermore, the computational requirements for training CFG-SegNet on extensive datasets highlighted the need for optimized computational strategies and resources to manage the substantial data processing demands effectively.

Our experiments find that CFG-SegNet trains more effectively on smaller patches than the entire CT image. A possible explanation is that minibatch discrimination in Age-ACP2P is a vital heuristic to maintain stability and diversity in image synthesis. Each training batch contains multiple 2D slices, and the generated segmentation mask from each piece is used to produce a 3D segmentation mask for 2.5D evaluation. The calculated 2.5D DSC averaged across four-fold cross-validation is then reported to assess our method.

Since our proposed method uses age information to improve segmentation performance, we divided the dataset into six groups based on the age of the subjects. Group 1 contains ages 1 to 3 years (infant), group 2 contains ages 4 to 6 years (preschool), group 3 contains ages 7 to 9 years (school-age I), group 4 contains ages 10 to 12 years (school-age II), group 5 contains ages 13 to 15 years (adolescent I), and group 6 contains ages of 16 years or more (adolescent II).

### 4.2. Segmentation Performance

For the quantitative evaluation, Table 1 summarizes the overall classification performance and shows the mean cross-validation segmentation results for four abdominal organs using CFG-SegNet and U-Net. The values shown in Table 1 show that CFG-SegNet significantly outperforms U-Net in segmentation accuracy. CFG-SegNet has an improved segmentation accuracy of 2.7% for the prostate, 2.6% for the uterus, 2.8% for the liver, and 3.4% for the heart. This indicates that CFG-SegNet is capable of achieving better segmentation accuracy by generating additional samples during training.

**Figure 3:**
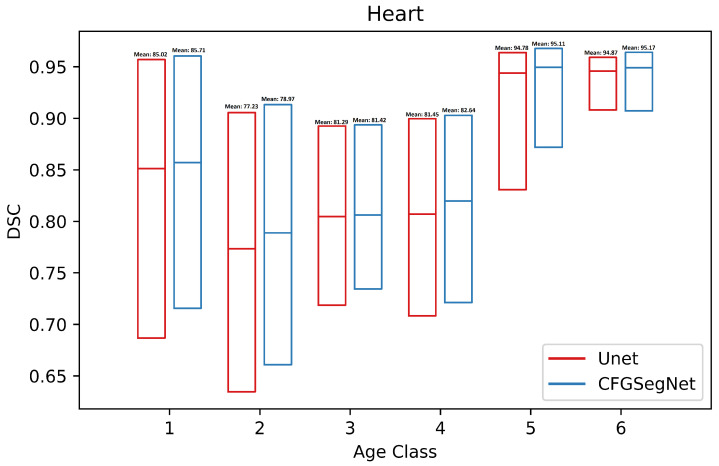
Paired class-wise boxplot of CFG-SegNet and U-Net for heart segmentation for six age classes (CFG-SegNet has a higher mean DSC in all age classes).

**Figure 4:**
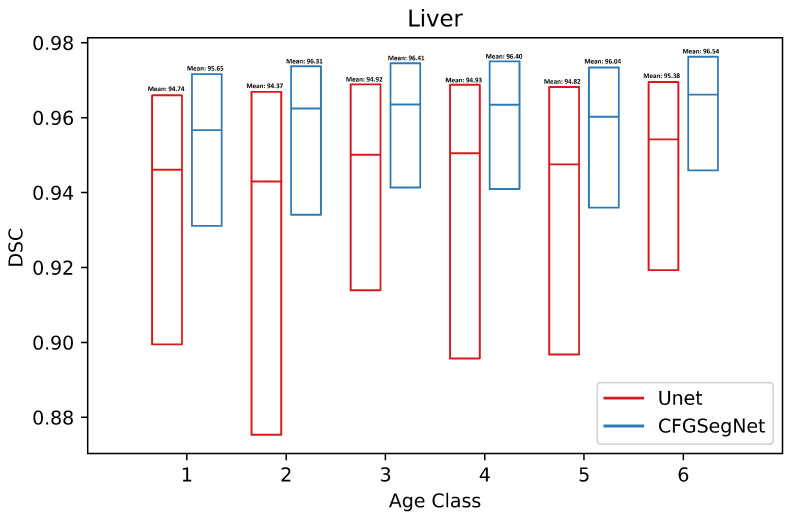
Paired class-wise boxplot of CFG-SegNet and U-Net for liver segmentation for six age classes (CFG-SegNet has a higher mean DSC in all age classes).

**Figure 5:**
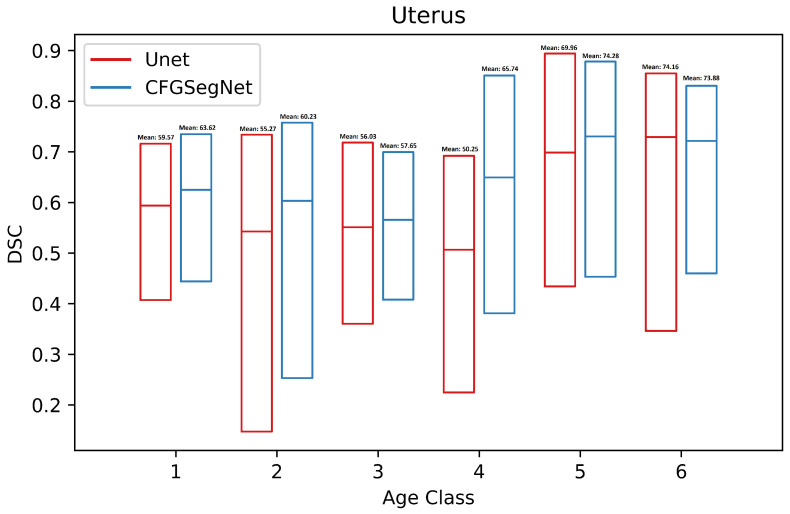
Paired class-wise boxplot of CFG-SegNet and U-Net for uterus segmentation six age classes (CFG-SegNet has a higher mean DSC in all age classes).

**Figure 6:**
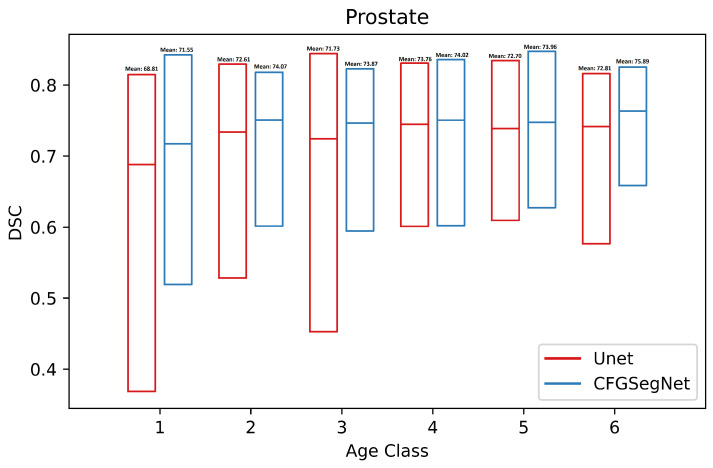
Paired class-wise boxplot of CFG-SegNet and U-Net for prostate segmentation for six age classes (CFG-SegNet has a higher mean DSC in all age classes).

In addition, the segmentation accuracy for each age class was calculated. Figure 3, Figure 4, Figure 5 and Figure 6 show a paired class-wise boxplot for each organ, which summarizes the segmentation results (DSC) of our proposed CFG-SegNet vs. U-Net across all six age groups. As shown in Figure 3, Figure 4, Figure 5 and Figure 6, CFG-SegNet achieved better segmentation results than U-Net across the six age classes for all four organs.

**Figure 7:**
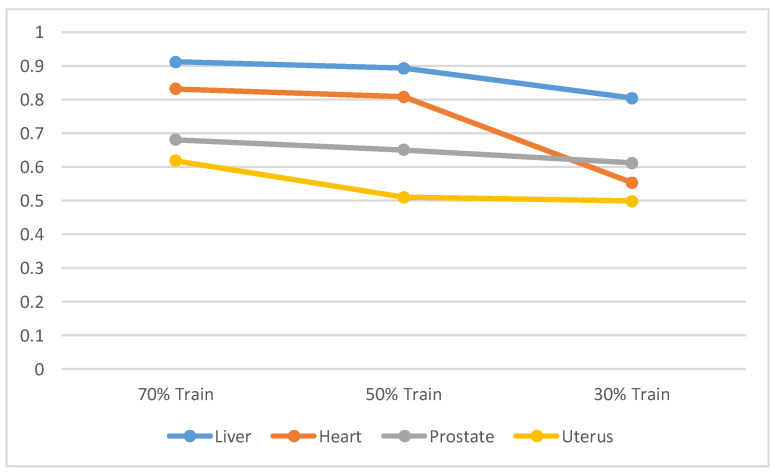
Conducted training experiments using varying percentages (30%, 50%, and 70%) of the available training samples.

In order to demonstrate the effectiveness and versatility of our methodology and to directly tackle the issue related to the volume of data required for training, we strategically conducted a series of experiments. In these experiments, our network was trained utilizing varying proportions of the available training dataset, specifically 30%, 50%, and 70%. The objective was to assess the performance and robustness of our approach under the conditions of limited data availability. The outcomes of these experiments, which highlight the capability of our method to maintain robust performance even when trained with a significantly reduced dataset, are depicted in Figure 7. This figure serves as a visual representation, providing compelling evidence of our method’s efficiency across different training scenarios.

**Figure 8:**
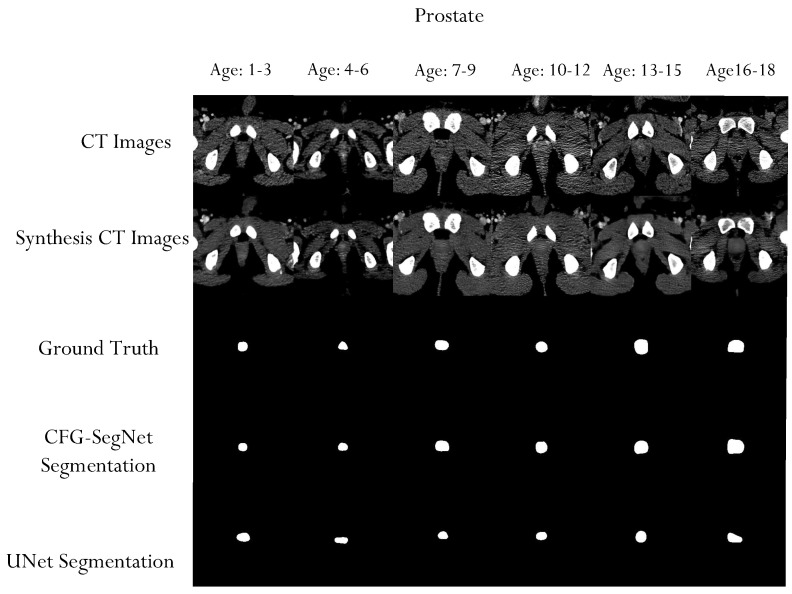
Sample prostate CT scans, ground truth masks, prostate CT synthesized images, and generated masks for each age group. The test images were conditionally synthesized, with a vector denoting the desired age classes. The synthesized prostate masks become elongated as the patient ages.

**Figure 9:**
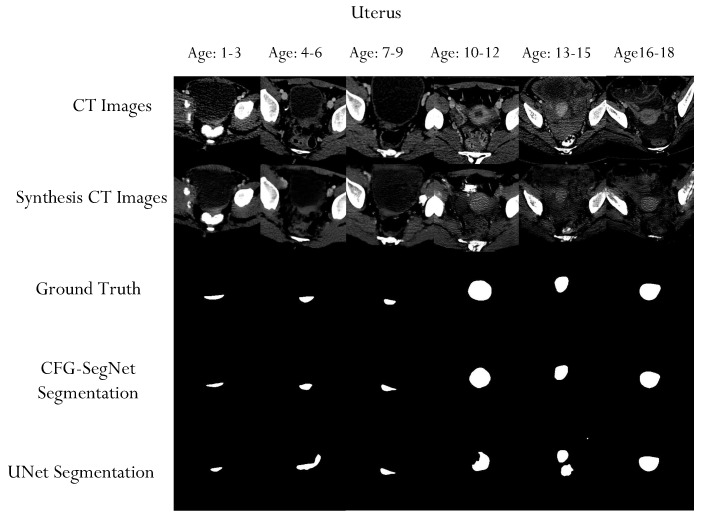
Sample uterus CT scans, ground truth masks, uterus CT synthesized images, and generated masks for each age group. The test images were conditionally synthesized, with a vector denoting the desired age classes. The synthesized uterus masks become elongated as the patient ages.

**Figure 10:**
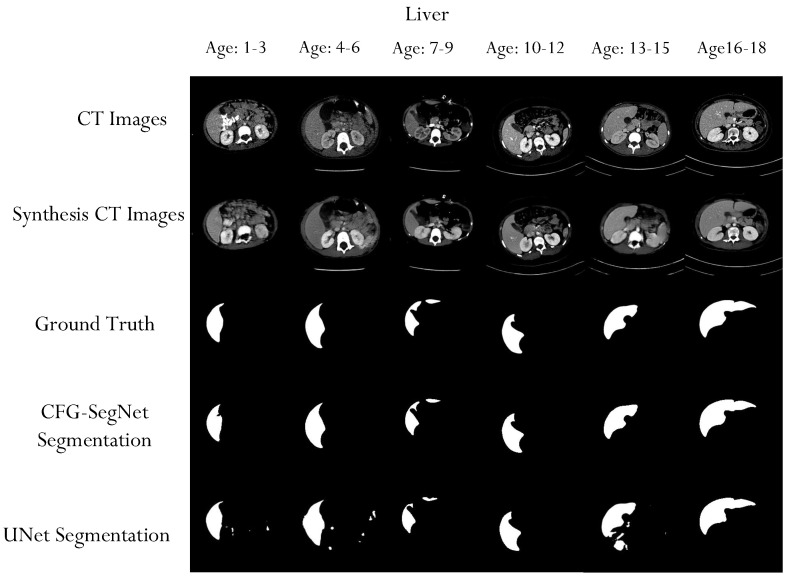
Sample liver CT scans, ground truth masks, liver CT synthesized images, and generated masks for each age group. The test images were conditionally synthesized, with a vector denoting the desired age classes. The synthesized liver masks become elongated as the patient ages.

**Figure 11:**
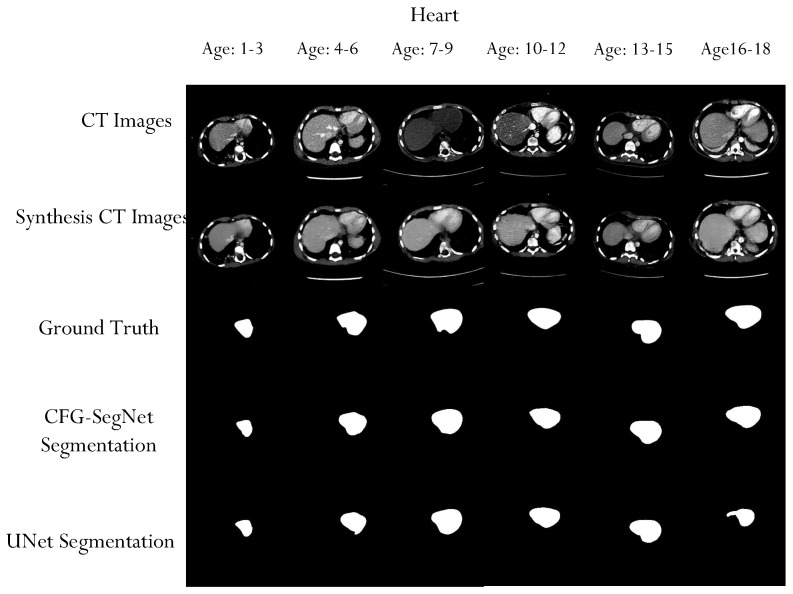
Sample heart CT scans, ground truth masks, heart CT synthesized images, and generated masks for each age group. The test images were conditionally synthesized, with a vector denoting the desired age classes. The synthesized heart masks become elongated as the patient ages.

A qualitative evaluation of our experiment also shows that the proposed method can generate high-quality organ segmentation masks. As shown in Figure 8, Figure 9, Figure 10 and Figure 11, the shapes of the masks generated by CFG-SegNet almost perfectly match the ground truth masks. The process of image synthesis was designed to reflect the physiological changes that occur in the organ as patients age. This is evidenced in the synthesized organ masks, which demonstrate a noticeable elongation in the structure with advancing age. Such changes are consistent with known patterns of prostate, uterus, liver, and heart growth and development over time, thus providing a realistic set of synthetic images for training and testing purposes. Additionally, Figure 8, Figure 9, Figure 10 and Figure 11 show that the segmentation masks generated by U-Net are of poorer quality than those generated by CFF-SegNet. This demonstrates CFG-SegNet’s ability to generate high-quality organ segmentation masks in CT images for classes with little training data. It is worth noting that synthesized training features with their generated masks for each age group are similar to the denoised versions of the original images, which is a common attribute of GAN-generated images. In addition, the use of geometric transformations as a baseline augmentation strategy for the UNet comparison group provides context for the sophistication and novelty of our synthetic image generation approach as an advanced form of data augmentation.

## 5. Conclusions

Accurately segmenting organs from CT scans is critical for clinical applications such as diagnostics, the progression of diseases over time, pre-operative planning, and dose estimation. Our work proposes and evaluates a novel hybrid medical image synthesis and organ segmentation framework. Our proposed framework uses an Age-ACP2P network conditioned on age, which generates training features during training to increase segmentation performance and accuracy. In addition, we propose a novel loss function that combines segmentation and adversarial losses and is used to train a conditional GAN and the segmentation network jointly. The main advantage of our proposed method is that our CFG-SegNet effectively addresses both the challenges of data imbalance and data limitations, all while maintaining high performance levels. In order to evaluate the efficacy of CFG-SegNet, we compared the segmentation results with the segmentation results from U-Net. In this experiment, we used the pediatric chest/abdomen/pelvic CT exam dataset, which has different organ contours. Our experimental results show our proposed method’s ability to better segment four different abdominal organs across six age classes compared to the U-Net alone.

## Figures and Tables

**Table 1:** Mean segmentation results for different organs with our proposed CFG-SegNet vs. U-Net. The values shown are the average results of the four-fold cross-validation experiment. The best results are highlighted in bold.

	Unet		CFG-SegNet
**Augmentation/Preprocessing**	-	**CutMix**	**Affine Transformations**
Liver	0.884±0.186	0.905±0.194	0.912±0.162
Heart	0.798±0.194	0.814±0.207	0.832±0.186
Prostate	0.654±0.257	0.669±0.222	0.681±0.252
Uterus	0.593±0.264	0.598±0.127	0.619±0.279

## Data Availability

The dataset used in this project was collected via the collaboration of researchers from Children’s Wisconsin, Marquette University, Varian Medical Systems, Medical College of Wisconsin, and Stanford University as part of a project funded by the National Institute of Biomedical Imaging and Bioengineering (U01EB023822). This dataset is developed for rapid, patient-specific CT organ dose estimation. The datasets [Pediatric Chest/Abdomen/Pelvic CT Exams with Expert Organ Contours (Pediatric-CT-SEG)] can be found here: https://wiki.cancerimagingarchive.net/pages/viewpage.action?pageId=89096588 (accessed on 10 October 2023).
